# Pre-procedural SARS-CoV-2 PCR testing in the pulmonary function laboratory at a tertiary government hospital in Qatar: A clinical audit

**DOI:** 10.5339/qmj.2022.fqac.26

**Published:** 2022-04-04

**Authors:** Fida Parveen, Merlin Thomas, Mansoor Hameed, Tasleem Raza, Balamurugan Panneerselvam, Rajalekshmi Nair, Mushtaq Ahmed, Irfan Ul Haq, Ahmed Al Mohammed, Hisham Abdul Sattar

**Affiliations:** ^1^Royal College of Surgeons in Ireland, Bahrain E-mail: MThomas27@hamad.qa; ^2^Pulmonary Division, Department of Medicine, Hamad Medical Corporation, Doha, Qatar; ^3^Weill Cornell Medical College, Qatar

**Keywords:** COVID-19, PFT, pre-procedural tests

## Abstract

Background: Prior to pulmonary function testing (PFT), local and international recommendations advise pre-procedural screening. Pulmonary function tests generate aerosol droplets containing millions of viruses, significantly increasing the risk of severe acute respiratory syndrome coronavirus 2 (SARS-CoV-2) transmission not only to the individuals in and around the PFT office, but also to subsequent patients who undergo the test later in the same room.

Methods: This clinical audit was carried out to establish the rate of positive pre-procedural SARS-CoV-2 PCR testing before a PFT. The data were obtained over a 6-week period from our ATS accredited pulmonary function laboratory at the Hamad General Hospital, Qatar (December 01, 2021, to January 10, 2022). The PFT laboratory was closed from January 10, 2022, till the date of this report (January 27, 2022) owing to an increase in COVID cases in the community in Qatar during the fourth wave.

Results: All the patients scheduled for PFT were asymptomatic of COVID-19. A total of 331 individuals were scheduled for PFT, and 221 PFTs were performed. There were 109 no-shows for both the PCR and the PFT. Between weeks 1 and 4, all the pre-procedural SARS-CoV-2 PCR tests were negative. The weekly average number of COVID-19 cases in Qatar increased from 157 per 100,000 population in week 1 to 2,918 in week 6.^2^ There was a similar trend in the pre-procedural SARS-CoV-2 PCR tests that increased and resulted in identifying 9 cases with positive SARS-CoV-2 PCR test over weeks 5 and 6 (Figure 1).

Conclusion: As the number of documented positive SARS-CoV-2 PCR tests in the community grew, so did the pre-procedural COVID-19 PCR positivity and the number of no-shows. The large number of no-shows may indicate greater worry or concern about contracting COVID-19 when visiting the hospital amid peak community cases. Our findings further call into question the utility of routinely performing pre-procedural PCR screening in asymptomatic cases when the prevalence of COVID-19 is low in the local population. Perhaps, it is time to consider replacing this with on-the-spot quick antigen testing for more effective use of resources.

## Figures and Tables

**Figure 1.**
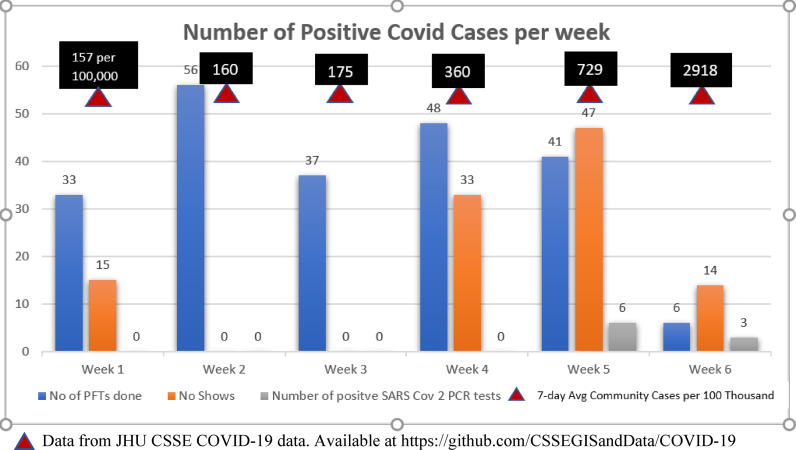
Bar chart showing weekly scheduled PFT cases, no-shows, SARS-CoV-2 PCR positive cases and weekly average community cases of COVID-19.
